# Economic Impact of an Employer-Sponsored Integrated Digital Intervention Targeting Substance Use Disorders: Return on Investment Analysis

**DOI:** 10.1089/tmj.2025.0102

**Published:** 2025-10-03

**Authors:** Suzette Glasner, Alfonso Ang, Darcy Michero

**Affiliations:** ^1^ Digital Therapeutics, Inc., New York, New York, USA.; ^2^ Department of Psychiatry, David Geffen School of Medicine, University of California, Los Angeles, California, USA.

**Keywords:** substance use disorder, return on investment, digital therapeutic, health economics, workforce, telemedicine

## Abstract

**Background:**

In light of the limited accessibility of evidence-based treatments for substance use disorders (SUD), coupled with the excess health care costs associated with untreated SUD, digital health has the potential to be transformative in addressing substance use and related chronic co-occurring conditions. This research aimed to evaluate the economic impact of Pelago (PEL), an integrated digital intervention combining psychosocial and pharmacological treatment targeting alcohol, tobacco, and opioid use disorders in a commercially insured adult population (N = 7,586). The model provided telehealth services using a smartphone application, from which clinician-facilitated videoconferencing and asynchronous messaging were delivered, along with digital, evidence-based therapy content and pharmacotherapy.

**Methods:**

Using a longitudinal model, a return on investment (ROI) analysis was undertaken to evaluate the impact of PEL on all-cause medical plan utilization costs 12 months before and after treatment initiation, relative to a matched control group.

**Results:**

In the 12 months following PEL registration, the intent-to-treat cohort who received the digital intervention evidenced an average all-cause medical plan utilization cost savings of $6,758 or 33% less per participant (4.5 ROI; p = 0.001). Among 1,172 participants who received PEL, a total savings of $7,920,376, relative to total program costs of $1,747,452 yielded a 4.5:1 ROI.

**Conclusions:**

Evidence-based treatment for SUD delivered via telehealth is associated with a significant positive ROI. Employers and payers willing to offer access to digital SUD care can mitigate morbidity and mortality while concurrently reducing medical costs and service utilization.

## Introduction

In light of robust evidence of the excess health care costs associated with substance use disorders (SUD)^1^ coupled with the observation that 70% of adults with SUD are in the workforce,^2^ it has become increasingly clear that employed American adults comprise the majority of SUD-related cost burden.^3^ Accordingly, the importance of quantifying these costs and developing cost-effective approaches to mitigating SUD-related morbidity and mortality has come into greater focus. This is particularly critical given that only 1% of workers receive treatment for SUD annually,^4^ and the costs associated with untreated SUD are disproportionately high.

Recent studies have made inroads to advance our understanding of medical costs associated with SUD, with the largest claims-based investigation to date revealing average annual costs directly attributable to SUD of $15,640 per affected individual and a total of $35.3 billion annually among employer-sponsored insured (ESI) adults.^1^ Moreover, this was deemed a likely underestimate, given that only 1% of the study population carried a SUD diagnosis, relative to the 13% of workers who self-report SUD.^5^

Alcohol and opioid use disorder (OUD) are the greatest drivers of health care utilization and excess costs across the SUD spectrum, accounting for $10.2 and $7.3 billion out of the total $35.3 billion in spending in attributable medical costs, respectively, in the ESI population.^1^ Likewise, given marked increases in inpatient and emergency care services linked with SUD by 44% in recent years,^6^ associated annual medical costs of these services were estimated to be over $13 billion, with the highest cost burden (∼$7.6 billion) incurred from alcohol-related disorders. Additionally, hospital encounters in which SUD is a complicating diagnosis have increased, with a quadrupling of OUD-related admissions between 1993 and 2016.^7^ Collectively, these findings highlight the urgent need to develop cost-effective ways to treat SUD.

Expanding access to evidence-based psychosocial and pharmacological SUD treatments at scale has proven challenging.^8–10^ Digital health interventions for SUD have the potential to bridge the sizable treatment gap that limits access to care to less than 10% of those who need it,^11^ particularly in under-resourced settings.^12^ Systematic reviews report promising clinical outcomes of digital interventions targeting various SUDs,^13–15^ and clinical studies underscore the safety, efficacy, and reduction in overdose mortality associated with telehealth-delivered medication treatment for OUD.^16,17^

One of the most notable gaps in the literature on digital health approaches to SUD treatment is the paucity of studies evaluating cost-effectiveness and return on investment (ROI).^18,19^ Such studies can have direct impacts on employers’ decision-making about SUD treatment investment. In turn, providing efficacious SUD care can reduce morbidity and mortality, expanding the reach of evidence-based treatments. As such, the primary aims of this study were to: (i) estimate all-cause medical plan utilization costs associated with SUD in an ESI population and (ii) evaluate the ROI associated with an integrated digital health intervention combining psychosocial and pharmacological treatment for adults with SUD.

## Methods

### STUDY DESIGN

This study was reviewed and exempted by the WCG institutional review board on the basis of criteria specified in Title 45 of the Code of Federal Regulations (45 CFR), Part 46.104. This longitudinal analysis evaluated the all-cause medical plan utilization costs in a commercially insured population, including medical conditions covered under health plan benefits, insurer, and study participant responsibility, relative to the cost of Pelago (PEL), an employer-sponsored, commercially available digital health intervention combining psychosocial and pharmacological treatment for SUD (i.e., the return on investment). To achieve this, PEL participants were matched to control group participants using demographic and SUD diagnostic data. A difference-in-differences (DID) quantitative approach compared medical costs in the 12 months before and post enrollment in PEL, relative to a matched control condition (CON). Using an intent-to-treat design, all participants who registered in PEL, irrespective of their level of treatment engagement, were included in the intervention group. Subsequently, sensitivity analyses were conducted to evaluate the stability of the ROI estimate.

### DIGITAL HEALTH INTERVENTION

PEL is an integrated digital health intervention combining psychosocial and pharmacological treatment targeting SUD through a HIPAA-compliant smartphone application. Cognitive behavioral therapy (CBT) and medication management visits are facilitated by a health coach (for those with tobacco use disorders [TUD]) or licensed drug and alcohol counselor and licensed prescriber, respectively, via telehealth video-based sessions, with concurrent access to medications for SUDs. The digital application contains interactive CBT and motivational therapy exercises, craving management tools, and substance use self-monitoring prompts. Messaging between participants and their coach or counselor in between sessions occurs asynchronously through a chat. PEL is described in detail in prior clinical trials.^20–22^

### SAMPLE

Eligible participants registered for PEL between January 1, 2021, and February 28, 2024. To be included in the study, individuals were: age ≥18, diagnosed with SUD (TUD, alcohol use disorder [AUD], or OUD), and continuously enrolled in their health plan at least 12 months before and 14 months following registration. Individuals were excluded from participation if they were missing demographic data or if they were not continuously enrolled in their health plan over the 26-month investigative period. Though exclusion of high-cost claimants was considered, to allow for natural variation in claims costs, the potential for outliers to confound outcomes was evaluated in sensitivity analyses. In the 12-month pre-enrollment period, baseline claims revealed a preexisting SUD diagnosis among 3.2% of the PEL group.

During PEL registration, participants provided informed consent digitally, allowing access to diagnostic, health care utilization, and medical cost data. Study participants (including those in the PEL and CON conditions) had a minimum of 24 months of continuous enrollment in their health plan (M = 29.1 months, SD = 3.26).

### DATA COLLECTION

Commercial health plan claims data were used to identify demographics, enrollment dates, diagnoses, procedures, and places of service, as well as amounts paid by both the plan members and the health plan for inpatient and outpatient medical care. The claims used in cost analyses were matched to study participants on the basis of the unique member identifier assigned to each individual. During data extraction, additional quality control measures employed to ensure that claims were not mismatched to spouses, children, or other dependents of members included cross-validation of claims with first and last name, date of birth, gender, and address data. Baseline costs were calculated on the basis of all expenditures incurred in the 12 months before PEL registration (or a matched index date for CON participants). Likewise, medical costs incurred in the 12 months following registration or corresponding index date for the PEL and control groups, respectively, comprised the post-intervention cost calculation. Listwise deletion was employed to ensure that only individuals with complete cost data were included in the final data set for analysis. Because of variability in data completeness, pharmacy costs were not included in the present analysis.

### COST ANALYSIS

#### Pre–post intervention cost variation analysis.

##### PROPENSITY SCORE MATCHING

To balance the distribution of covariates between PEL and CON, propensity score matching was conducted (Stuart, 2010) using a logistic regression model. Propensity scores were calculated for each study participant using three variables: age, sex, and SUD (tobacco, alcohol, and opioid); subsequently PEL participants were matched one-to-one without replacement to CON participants by propensity score, age, and sex. To ensure that this process effectively mitigated any imbalance in potential covariates between groups, *t-*tests were used in analyses of continuous variables, with chi-squared tests for categorical variables. All analyses were conducted using SAS version 9.4 (SAS Institute, Cary, NC).

##### DID DESIGN

The DID approach was used to examine the average changes in all-cause medical plan utilization costs from the baseline to the post-intervention period as a function of the group. To achieve this, the average costs incurred during the 12-month baseline period were subtracted from those incurred over the 12 months following registration (or matched index date) for PEL and CON participants, respectively. Next, the pre- to post-intervention period cost differences were compared between groups using a General Linear Model. Finally, the DID (i.e., average cost savings) was calculated by subtracting the mean pre- to postintervention difference in medical costs in the CON group from that observed in the PEL group, after adjusting for baseline differences between groups.

#### Program fees and ROI analysis

PEL fees were billed as an annual case rate for access to 12 months of SUD care. The fees varied depending upon the intensity and scope of services provided. A weighted average fee per participant was calculated, taking into account the number of participants in each of three intensity tiers, which comprised the following services in order from lowest to highest intensity: (1) supportive or preventive services (i.e., health coaching, access to digital evidenced-based behavioral therapy content, remote monitoring and asynchronous support through a chat function within the PEL smartphone application, pharmacotherapy for nicotine cessation), (2) behavioral therapy services targeting SUD (i.e., evidence-based counseling provided via telehealth by a state-licensed drug and alcohol specialized clinician weekly based on clinical needs, access to digital evidence-based therapy content, remote monitoring and asynchronous counseling), and (3) behavioral therapy services as described in (2) combined with pharmacotherapy for alcohol and/or OUD and medical management provided via telehealth monthly or more frequently depending on clinical needs, by a state-licensed prescribing provider (i.e., physician or nurse practitioner) ROI was calculated by dividing the cost savings by the average fee per participant.

### SENSITIVITY ANALYSIS

To evaluate the stability of the ROI estimate, sensitivity analyses examined the impact of variation in parameters that could potentially influence cost savings.

## Results

Between January 1, 2021, and February 28, 2024, 1,172 participants who registered for PEL met the inclusion criteria for the study. These participants were matched to 6,414 CON participants. Distributions of baseline covariates including age, gender, and geographic region were similar between the PEL and control groups (*Table 1*), with no significant differences observed.

**Table 1.** Baseline Characteristics of Participants

	PEL (*N* = 1,172)	CONTROL (*N* = 6,414)	*p*
Sex (female), *n* (%)	580 (49.5%)	3,188 (49.7%)	0.89
Age (mean, SD)	45.1 (SD = 11.6)	45.4 (SD = 10.9)	0.39
Substance use disorder, *N* (%)	1,172 (100%)	6,414 (100%)	0.95
Tobacco	552 (47.1%)	3,091 (48.2%)	
Alcohol	546 (46.6%)	2,893 (45.1%)	
Opioid	74 (6.3%)	430 (6.7%)	
Region, *N* (%)			
Midwest	143 (12.2%)	1,661 (25.9%)	0.98
Northeast	308 (26.3%)	776 (12.1%)	
South	466 (39.8%)	2,530 (39.4%)	
West	145 (12.4%)	814 (12.7%)	
Missing	110 (9.4%)	633 (9.9%)	
Baseline all-cause medical plan utilization costs, mean (SD)	$11,920 (9,862)	$16,196 (12,523)	<0.001

Statistically significant differences were defined by alpha level *p* < 0.05.

PEL, Pelago.

### COST ANALYSIS

Because PEL evidenced lower average baseline all-cause medical plan utilization costs relative to CON (M = $11,920 versus M = $16,196, respectively, *p* < 0.001), in ROI analyses and cost savings calculations, all outcomes adjusted for these baseline group differences. In addition, to evaluate the potential impact of educational attainment, socioeconomic status, and co-occurring chronic disease status on baseline costs, retrospective group comparisons on these variables were conducted. The proportion of individuals in the PEL versus CON groups who attained high school education did not differ significantly (92.6% vs. 92.9%, *p* = 0.31), nor did the median household income level (M = $117,003 ± $44,505 vs. M = $118,209 ± $46,593, *p* = 0.63). Based on guidance from prior work establishing the prevalence of multiple chronic conditions,^23^ health status was defined by the presence of one or more of the following: arthritis, COPD, coronary heart disease, diabetes, hepatitis, hypertension, stroke, and kidney disease. The groups were found to be comparable in terms of health status, with 41.9% of those in PEL evidencing chronic conditions versus 40.1% of CON participants (χ^2^ <1, df = 1, *p* = 0.45).

### PRE–POST INTERVENTION COST VARIATION ANALYSIS

When compared to CON, individuals in PEL evidenced lower all-cause medical plan utilization expenditures by 33% in the 12 months postregistration relative to the baseline year. *Fig. 1* depicts the changes in costs for the two groups over the 24-month study period.

**Fig. 1.**
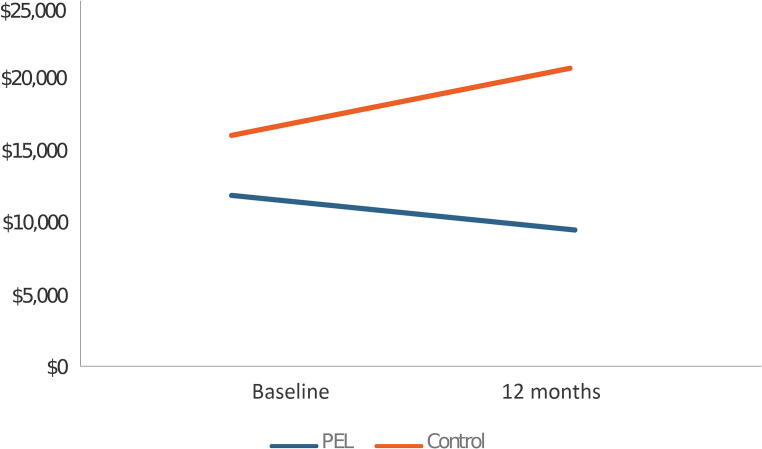
Changes in all-cause medical plan utilization costs by group.

### DID COST SAVINGS OUTCOMES

General linear modeling was used to compare all-cause medical plan utilization costs among individuals in the PEL group relative to propensity-matched CON participants. Those in PEL had significantly lower average health care expenditures (DID = $6,758, *p* < 0.001) in the 12 months postregistration, compared to CON (*Table 2*). When examined separately according to primary SUD, those with AUD (*n* = 546) evidenced the largest magnitude of savings (DID = $7,305, *p* < 0.001) relative to matched controls, followed by TUD (DID = $6,299, *p* < 0.001) and OUD (DID = $6,147, *p* < 0.001).

**Table 2.** Changes in Inpatient and Outpatient All-Cause Medical Plan Utilization Costs by Group

	ALL-CAUSE MEDICAL PLAN UTILIZATION COSTS					OUTPATIENT COSTS					INPATIENT COSTS				
	MEAN BASELINE COST	MEAN POST-12 MONTH COST	DIFF	DID	*p*	MEAN BASELINE COST	MEAN POST-12 MONTH COST	DIFF	DID	*p*	MEAN BASELINE COST	MEAN POST-12 MONTH COST	DIFF	DID	*p*
PEL CON	$11,920$16,196	$9,545$20,579	$2,375 −$4,383	$6,758	<0.001	$4,887$6,316	$3,581$7,654	−$1,306$1,338	$2,644	<0.001	$5,483$7,288	$3,704$8,416	−$1,779$1,128	$2,907	<0.001

CON, control group; DID, difference-in-differences; Diff, difference; PEL, digital health intervention condition.

### PROGRAM FEES AND ROI ANALYSIS

ROI was calculated by dividing the average cost savings, determined by the DID methodology, by the average weighted PEL program fee ($1,491). As described in *Table 3*, the ROI overall was 4.5, with the TUD cohort achieving the highest ROI (14.6:1, *p* < 0.001), followed by the AUD (3.1:1, *p* < 0.001) and OUD cohorts (2.0:1, *p* < 0.001). The total savings attributed to the PEL group was $7,920,376 ($6,758 × PEL sample size 1,172), with the AUD, TUD, and OUD cohorts realizing, $3,988,530, $3,476,968, and $454,878 in cost savings, respectively.

**Table 3.** Return on Investment

	OVERALL SAMPLE	OPIOID USE DISORDER	ALCOHOL USE DISORDER	TOBACCO USE DISORDER
Participants (*N*)	1,172	74	546	552
Total medical cost savings	$7,920,376	$454,878	$3,988,530	$3,476,968
Total PEL program costs	$1,747,753	$227,352	$1,282,346	$238,055
ROI	4.5	2.0	3.1	14.6

ROI, return on investment.

#### Cost savings drivers and mechanisms of action

The proportion of cost savings accounted for by inpatient versus outpatient services was nearly evenly split, with 43% of savings allocated as inpatient service costs ($2,907, *p* < 0.001), and 39% as outpatient costs ($2,644, *p* < 0.001); both categories of service were lower in spend for the PEL group (*Tables 2* and *4*). Moreover, the frequency of inpatient admissions in the 12 months post-baseline was 31% lower among PEL, relative to CON (*p* < 0.001). Likewise, utilization of emergency room and urgent care services was 29% lower at 12 months post-baseline relative to those in CON (*p* < 0.001) (*Table 4*).

**Table 4.** Mean Differences in All-Cause Medical Plan Utilization Costs from Baseline to 12 Months Separated by Places of Service, Between PEL and Control Groups

PLACE OF SERVICE	MEAN BASELINE	MEAN 12-MO	DIFF	DID	% SAVINGS (%)
Inpatient					
PEL	$5,483.20	$3,704.41	−$1,778.79	$2,907.40	43.0
CON	$7,288.20	$8,416.81	$1,128.61		
Outpatient					
PEL	$4,887.20	$3,581.28	−$1,305.92	$2,644.86	39.1
CON	$6,316.44	$7,655.39	$1,338.95		
Ambulatory Surgical Center					
PEL	$1,311.20	$954.50	−$356.70	$754.17	11.2
CON	$1,813.95	$2,211.42	$397.47		
ER/urgent care					
PEL	$441.04	$334.08	−$106.97	$248.86	3.7
CON	$592.77	$734.67	$141.90		
Other					
PEL	$369.52	$276.81	−$92.72	$202.74	3.0
CON	$502.08	$612.10	$110.03		
				$6,758	100.0

*Tables 5* and *6* summarize indicators of PEL engagement. To evaluate the relationship of treatment engagement to medical costs among those with AUD and OUD, a composite score was employed, summing two core engagement behaviors: total PEL appointments attended and total app/digital engagement activities. The total digital engagement score was used to evaluate the engagement-cost relationship for those with TUD, given the emphasis on digital interactions in the TUD treatment model, contrasted with a focus on clinical appointments for those with AUD and OUD. For the combined AUD+OUD cohort, the mean overall engagement score was 37.5 (SD = 86.7). Highly engaged participants were defined as those with a composite score ≥60. This cutoff value was determined using logistic regression coupled with examining the value of the area under the receiver operating curve (ROC). The optimal cut-point value occurs when the sensitivity and specificity approximate the value of the area under the ROC curve and the absolute value of the difference between the sensitivity and specificity values is minimized.^24–26^ Engagement was associated with cost savings; medical costs were an average of $2,122 lower among the highly engaged group (*t*[619] = 2.01, *p* = 0.04), relative to the less engaged group. However, cost differences in relationship to digital app engagement among those with TUD did not achieve significance.

**Table 5.** PEL per Participant Engagement Among Individuals with Alcohol and Opioid Use Disorders

	ALCOHOL USE DISORDER M (SD)	OPIOID USE DISORDER M (SD)	TOTAL M (SD)
Counseling appointments attended	8.3 (11.8)	8.1 (14.9)	8.3 (12.2)
Provider appointments attended	1.6 (4.2)	7.1 (12.4)	2.2 (5.9)
Total appointments attended	9.9 (14.4)	15.2 (26.1)	10.5 (16.1)
Digital CBT steps completed	5.0 (8.9)	2.4 (6.4)	4.7 (8.8)
Digital check-ins	28.9 (76.3)	26.8 (76.5)	28.7 (76.3)
Total app engagement activities	76.9 (152.5)	95.0 (229.7)	79.0 (163.0)
Total participant sent messages	8.1 (30.6)	18.3 (50.4)	9.2 (33.6)
Total messages sent	17.3 (52.2)	35.8 (91.8)	19.4 (58.3)

Provider refers to a physician or nurse practitioner who facilitates medication management visits.

CBT steps = digital CBT skills modules with interactive therapeutic exercises. Digital check-ins = substance use self-monitoring logged into the app. Participant and total sent messages = asynchronous chat communication between participant and counselor.

CBT, cognitive behavioral therapy.

**Table 6.** PEL Per Participant Engagement Among Individuals with Tobacco Use Disorders

	TOBACCO USE DISORDER M (SD)
CBT steps completed	15.8 (23.4)
Digital check-ins	9.7 (27.9)
Total app engagement activities	71.5 (108.5)
Total member sent messages	8.4 (14.7)
Total messages sent	22.8 (33.7)

CBT steps = digital CBT skills modules with interactive therapeutic exercises. Digital check-ins = tobacco use self-monitoring logged into the app. Member and total sent messages = asynchronous chat communication between participant and coach.

Diagnostic drivers of ROI were subsequently examined (*Table 7*). The majority of savings fell into the SUD category (15.84%, $1,254,588), followed by psychiatric (2.79%, $220,978) and gastrointestinal (2.01%, $159,200).

**Table 7.** Diagnostic Drivers of Cost Savings at 12 Months Postbaseline

	% OF OVERALL COST FOR PELAGO GROUP (%)	% OF OVERALL COST FOR CONTROL GROUP (%)	DIFFERENCE BETWEEN GROUPS (%)
Cancer	5.02	6.81	1.79
Gastrointestinal	7.41	9.42	2.01
Musculoskeletal	7.08	8.58	1.50
Substance use disorder	7.72	23.56	15.84
Cardiovascular	7.42	8.77	1.35
Neurological	4.10	4.95	0.85
Pulmonary	3.45	4.87	1.41
Infectious diseases	2.08	2.28	0.20
Urinary and kidney	1.81	1.86	0.06
Psychiatric	4.11	6.90	2.79
Endocrine	3.27	3.39	0.12
Dermatology	0.50	1.38	0.88
Obstetrics and gynecology	0.66	0.70	0.04
Other diseases	5.99	6.09	0.10
Immune system	0.02	0.29	0.27
Metabolic	0.36	0.38	0.02

### SENSITIVITY ANALYSIS

To evaluate the potential impact of higher baseline costs among CON participants on overall ROI, sensitivity analyses were performed. Using *Z*-scores, outliers exceeding 4 standard deviations from the median (∼$200,000) were removed; this analysis reduced the baseline difference between PEL and controls slightly (PEL = $11,944 vs. CON = $14,771). The resulting ROI was largely unchanged from the original analysis (ROI = 4.54, DID = $6,772, *p* < 0.001). Next, outliers with 1.5 times the interquartile range were removed; the ROI increased slightly as a result (ROI = 4.58, DID = $6,830, *p* < 0.001), despite narrowing the baseline cost differences (PEL = $11,944 vs. CON = $15,062).

## Discussion

Given the magnitude of the public health impact of SUD, scalable interventions are urgently needed to reverse the opioid overdose crisis and minimize the detrimental impacts of substance use on health and longevity.^27,28^ Evidence-based treatments for SUD are vastly underutilized due to high costs of care, limited availability of specialty training, regulatory restrictions on medication treatments, stigma, and systemic barriers.^29–31^ Employers are well positioned to make an impact on SUD care access, given the high proportion of affected individuals who are in the workforce. Importantly, understanding the cost–benefit of doing so is essential to facilitate employers’ prioritization of addressing SUD. To our knowledge, the present investigation is the first to evaluate the ROI of a digital health intervention for SUD in an ESI population. Our findings demonstrate that the virtual delivery of evidence-based SUD treatments yields a significant ROI, reducing all-cause medical plan utilization spending by 33%. PEL produces clinically meaningful changes in tobacco,^22,32,33^ alcohol,^21^ and opioid use.^17,20,34^ In the present study, PEL was associated with a $6,758 per participant reduction in health care costs, yielding a 4.5:1 ROI over the first year. The ROI differed moderately between the OUD and AUD cohorts, ranging from 2:1 to 3.1:1, with both differing notably from the TUD group, for whom a 14.6:1 ROI was demonstrated. This was likely due largely to lower program costs, as the weighted TUD program fee was $431, nearly 3.5 times less costly than the weighted fee of $1,491 across programs.

Though a handful of studies have evaluated the cost-effectiveness of digital health interventions for SUDs,^35,36^ this literature focuses primarily on web- or telephone-based CBT interventions,^37,38^ whereas mobile phone applications are the most ubiquitous platform for digital health care delivery. Methodologically, extant studies have not reported ROI, but rather characterize the difference in costs between digital and traditional face-to-face interventions, and the potential to avoid high-cost health service utilization,^39^ relying largely on mathematical models rather than real-world data to estimate medical costs.^40,41^ The present investigation extends these findings, in quantifying savings associated with changes in both inpatient (43%) and outpatient (39%) service utilization. Notably, the reductions in ER visits and inpatient admissions by nearly one-third in this study are consistent with prior work demonstrating lower rates of acute care associated with telehealth-delivered SUD treatment.^15,42^

Though it is well established that engagement is a robust predictor of clinical outcomes for those with SUD,^43^ this is the first study to describe an association between engagement in digitally delivered SUD care and claims-based medical costs. While these analyses were exploratory in nature, the observation that engagement with both clinician- and digital app-based PEL intervention components is associated with costs suggests that developing innovative methods to promote engagement in digital therapeutic interventions is a promising area of future investigation.

Several limitations of this study warrant comment. First, the baseline difference in medical costs between PEL and CON was significant. Though the impact of this difference on cost-effectiveness was mitigated by adjusting cost savings estimates accordingly, the use of DID methodology for evaluating ROI, and sensitivity analyses demonstrating the stability of the ROI estimate when baseline cost parameters varied, the reason for the elevated baseline costs in the control group warrants further investigation. One possible explanation is that matching control group members to the PEL cohort based on SUD diagnosis uncovered individuals who were experiencing health-related impacts of SUD, suggesting the presence of longstanding or severe SUD in the control group. On the other hand, only 3.2% of those in PEL had a documented SUD diagnosis during the baseline period, suggesting that they presented for care earlier in the course of their SUD. Because of variability in SUD severity specifiers in the claims data, it was not possible to directly compare the distribution of SUD severity between the PEL and control groups, an important area of future research.

Additionally, though the groups were found to be comparable in demographics, socioeconomic status, and the presence of overlapping chronic diseases, data concerning workplace characteristics (e.g., salaried vs. nonsalaried employment status) were not available and therefore, their impact on the overall health and baseline costs of those who accessed PEL versus controls could not be evaluated. Though all individuals in the medically enrolled population under study had access to PEL, the influence of workplace characteristics on the utilization of PEL is an important area of future investigation. Second, because of incomplete access to pharmacy claims, we were unable to account for this element of medical costs. Third, because the study focused on an ESI population, the findings may not be an accurate representation of potential cost savings for individuals without health insurance or who receive insurance that is not employer-sponsored. That said, the decision not to match the groups on baseline costs was intentional to optimize the generalizability of the findings.

Finally, because of the need to examine smaller subgroups to evaluate participant engagement, the analysis was underpowered; future studies could extend the present engagement analysis by further specifying the digital and clinician-facilitated engagement activities that impact costs and improve health outcomes.

## Conclusions

In demonstrating a significant ROI and describing the diagnostic and health service utilization mechanisms underlying cost savings, this study fills a critical gap in the literature on digital health interventions for SUD. While the potential cost-effectiveness of virtual care approaches has been touted as a major potential advantage of telemedicine and other technology-assisted treatment modalities, evaluating this hypothesis with real-world health claims data generates a pathway to action. Employers and payers are confronted with the need to prioritize benefit offerings often in the absence of a comprehensive data-based representation of population needs. The present results demonstrate that digital health interventions have the potential to reduce costs and health service utilization and improve overall health among individuals with SUD.
